# How is atrial fibrillation detected in everyday healthcare? Results of a Dutch cohort study

**DOI:** 10.1007/s12471-022-01719-2

**Published:** 2022-09-01

**Authors:** N. Verbiest-van Gurp, S. B. Uittenbogaart, S. C. M. van de Moosdijk, U. F. van Sprang, J. A. Knottnerus, H. E. J. H. Stoffers, W. A. M. Lucassen

**Affiliations:** 1 Department of Family Medicine, Care and Public Health Research Institute, Faculty of Health, Medicine, and Life Sciences, Maastricht University, Maastricht, The Netherlands; 2 Department of General Practice, Amsterdam Public Health, Amsterdam University Medical Centres, University of Amsterdam, Amsterdam, The Netherlands

**Keywords:** Atrial fibrillation, Diagnosis, General practice, Cardiology, Electrocardiography, Aged

## Abstract

**Background:**

Atrial fibrillation (AF) is a common arrhythmia with serious potential consequences when left untreated. For timely treatment, early detection is imperative. We explored how new AF is detected in patients aged ≥ 65 years in Dutch healthcare.

**Methods:**

The study cohort consisted of 9526 patients from 49 Dutch general practices in the usual-care arm of the Detecting and Diagnosing Atrial Fibrillation study. We automatically extracted data from the electronic medical records and reviewed individual records of patients who developed AF. Patient selection started in 2015, and data collection ended in 2019.

**Results:**

We included 258 patients with newly diagnosed AF. In 55.0% of the patients, the irregular heartbeat was first observed in general practice and in 16.3% in the cardiology department. Cardiologists diagnosed most cases (47.3%), followed by general practitioners (GPs; 33.7%). AF detection was triggered by symptoms in 64.7% of the patients and by previous stroke in 3.5%. Overall, patients aged 65–74 years more often presented with symptoms than those aged ≥ 75 years (73.5% vs 60.6%; *p* = 0.042). In 31.5% of the patients, AF was diagnosed incidentally (‘silent AF’). Silent-AF patients were on average 2 years older than symptomatic-AF patients. GPs less often diagnosed silent AF than symptomatic AF (21.0% vs 39.0%; *p* = 0.008), whereas physicians other than GPs or cardiologists more often diagnosed symptomatic AF than silent AF (34.6% vs 11.9%; *p* < 0.001). Most diagnoses were based on a 12-lead electrocardiogram (93.8%).

**Conclusion:**

Diagnosing AF is a multidisciplinary process. The irregular heartbeat was most often detected by the GP, but cardiologists diagnosed most cases. One-third of all newly diagnosed AF was silent.

**Supplementary Information:**

The online version of this article (10.1007/s12471-022-01719-2) contains supplementary material, which is available to authorized users.

## What’s new?

- Two-thirds of the patients were diagnosed with atrial fibrillation (AF) based on their symptoms, whereas one-third had silent AF.
- General practitioners (GPs) were often the first to detect an irregular heartbeat, whereas cardiologists most often diagnosed AF.
- Diagnosing new AF is often a multidisciplinary process, in which not only cardiologists but also GPs and other physicians are frequently involved.
- Almost all new AF diagnoses were based on a 12-lead electrocardiogram.

## Introduction

Atrial fibrillation (AF) is a common arrhythmia among the elderly and is associated with considerable comorbidity [1–3]. Up to 25% of ischaemic strokes are related to AF [4, 5]. Since adequate antithrombotic treatment reduces stroke risk in AF patients by 60%, early detection of AF is crucial [6]. However, detection of AF can be challenging. Approximately one-third of patients have no symptoms (‘silent AF’) [7, 8]. Without symptoms, patients do not seek medical attention and physicians are not triggered to perform diagnostic tests. Silent AF can be discovered incidentally, for example when measuring the blood pressure or through screening. Paroxysmal AF further complicates detection because of its intermittent character. As a result, AF may remain undetected.

Exploring current clinical practice could uncover possible strategies to improve AF detection. In Dutch healthcare, all inhabitants are registered with a general practitioner (GP). In case of health-related issues, this physician is consulted first. Outside of office hours, patients can contact the out-of-hours primary care service. As not all general practices and out-of-hours services have a 12-lead electrocardiogram (*ECG*) device [9], some GPs have to refer their patients to the cardiologist to confirm AF. Previously, we have investigated AF detection by Dutch GPs and cardiologists in two case vignette studies [10, 11]. GPs indicated to have adequate equipment, knowledge and experience to detect and diagnose AF, whereas cardiologists reported having access to a wide variety of diagnostic tools. Most GPs and cardiologists chose a shorter monitoring duration than AF guidelines recommend for patients with symptoms occurring less than once daily [12, 13].

In the present cohort study, we investigated how AF is detected in patients aged ≥ 65 years in everyday Dutch healthcare. We examined what triggered the detection of AF, where the irregular heartbeat was first noticed, who diagnosed AF and which diagnostic devices were used.

## Methods

### Study design and setting

This study included data from the usual-care control arm of the Detecting and Diagnosing Atrial Fibrillation (D_2_AF) study, a cluster-randomised controlled trial comparing opportunistic screening for AF with usual care [14, 15]. Participating practices (*n* = 49) were evenly distributed across the Netherlands (see Figure S1 in the Electronic Supplementary Material).

### Participants and data extraction

Patients were selected from October 2015 through September 2017. For each practice, we randomly selected 200 electronic medical records of patients aged ≥ 65 years without an International Classification of Primary Care (ICPC) code for AF. In one small practice, only 189 patients met these criteria, bringing the total to 9789 patients. To avoid the observer’s paradox (i.e. influencing usual care regarding AF detection due to awareness of the study), both patients and healthcare workers were unaware of who had been selected. We extracted baseline characteristics from the electronic medical records of the study cohort from May 2018 through January 2019. Follow-up time differed per practice.

To identify all newly diagnosed AF cases after the study period, we manually reviewed all patient records with ICPC codes for AF, palpitations, paroxysmal tachycardia, ectopic heartbeats, other abnormal heartbeats, transient ischaemic attack or stroke. We considered AF confirmed if the AF was recorded on a 12-lead ECG, Holter monitor or event recorder. Recording time was not registered. Patients with atrial flutter were also included, as atrial flutter has the same ICPC code as AF, can cause the same symptoms, can convert into AF and also requires antithrombotic treatment.

### Data collection

We entered pseudonymised data of patients with newly diagnosed AF in a cloud-based electronic case report form (Castor Electronic Data Capture, Ciwit BV, Amsterdam, the Netherlands) using checkboxes and free text. To track what triggered the AF diagnosis, where the irregular heartbeat was detected, which medical professional diagnosed AF and which diagnostic tests were performed, we reviewed the following sections of the medical record: journal, medical history, discharge letters, outpatient letters and medication overviews. In case of any doubt, data collectors (SU, SvdM, UvS, KC, YG) reached consensus through discussion.

### Research ethics

The medical ethics board of the Amsterdam University Medical Centres approved the current study in an amendment to the original study protocol (NL48215.018.14), which is registered in the Netherlands Trial Register (identification number: NL4776; previously: NTR4914).

### Data analysis

First, we compared baseline characteristics of patients who did or did not develop AF. Second, we analysed gender- and age-related differences in the trigger for AF diagnosis, i.e. previous stroke, suspect symptoms or incidentally. Third, we compared patients in whom the AF diagnosis was made incidentally (silent AF) with those in whom the diagnosis was made following examination based on the presence of suspect symptoms or a previous stroke (symptomatic AF).

Continuous variables were compared with independent-sample *t*-tests. We compared categorical variables with a chi-square test and used Fisher’s exact and Fisher-Freeman-Halton exact tests where appropriate. Free-text comments were categorised by theme. Categorical data are presented as number (%) and numerical data as mean ± standard deviation (SD). A *p*-value < 0.05 was considered statistically significant. We used IBM SPSS 25 Statistics to perform the analyses.

## Results

### Study population

**Fig. 1:**
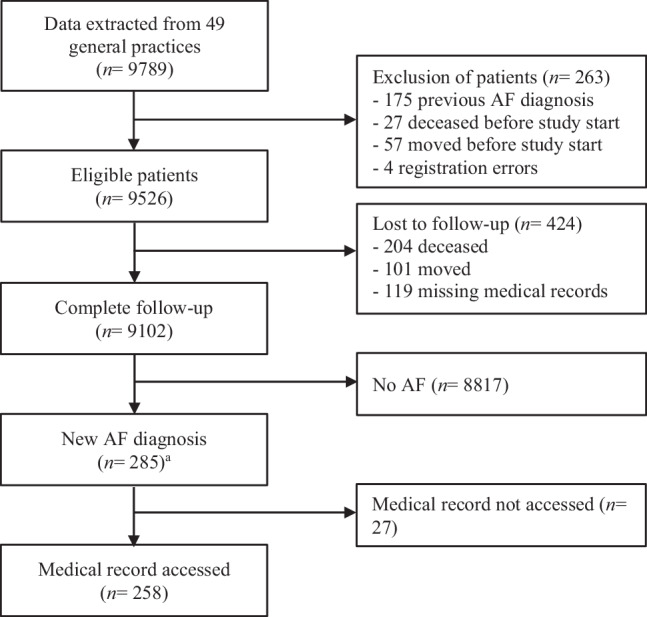
Follow-up of study cohort. ^a^Due to a longer follow-up time, more patients were diagnosed with new atrial fibrillation (*AF*) in the current study (*n* = 285) than in the original cluster-randomised controlled trial (*n* = 139)

We included 49 general practices and formed a study cohort of 9526 patients (Fig. 1), of whom 285 (3.0%) had newly diagnosed AF. On average, patients with AF were older and more often had diabetes mellitus, heart failure, hypertension or vascular disease than those without AF (Tab. 1).

**Table 1:** Baseline patient characteristics, stratified by new atrial fibrillation (*AF*) diagnosis

Variable	Patients (*N* = 9526)	New AF (*n* = 285)	No AF (*n* = 9241)	*P*-value
Age in years	75.0 ± 6.9	77.6 ± 7.1	74.9 ± 6.9	< 0.001
Female	5177 (54.3)	148 (51.9)	5029 (54.4)	0.406
*Comorbidity*^a^	6080 (64.7)	215 (75.4)	5865 (64.4)	< 0.001
– Diabetes mellitus	1750 (18.6)	77 (27.0)	1673 (18.4)	< 0.001
– Heart failure	362 (3.9)	25 (8.8)	337 (3.7)	< 0.001
– Hypertension	4579 (48.7)	170 (59.6)	4409 (48.4)	< 0.001
– Previous stroke/TIA	911 (9.7)	36 (12.6)	875 (9.6)	0.089
– Thromboembolism	431 (4.6)	15 (5.3)	416 (4.6)	0.579
– Vascular disease^b^	1573 (16.7)	62 (21.8)	1511 (16.6)	0.021

Data are mean ± standard deviation or *n* (%)

*TIA* transient ischaemic attack

^a^Data on comorbidity were missing for 127 patients (all of whom were in the no-AF group)

^b^Vascular disease includes peripheral vascular disease, myocardial infarction and angina pectoris

Mean time between defining the study cohort and reviewing the medical records was 25.6 ± 5.8 months. We were able to review the medical records of 258 patients with AF, of whom 23 had atrial flutter. Women were diagnosed with AF at an older age than men (79.7 vs 77.5 years; *p* = 0.014). Mean CHA2DS2-VASc score for AF patients was 3.7 ± 1.5. Women had a higher mean CHA2DS2-VASc score than men (4.2 vs 3.2; *p* < 0.001), probably because this score assigns 1 point for female gender.

### Trigger for AF detection

Symptoms triggered AF detection in 167 patients (64.7%), while stroke was the trigger in 9 patients (3.5%) (Tab. 2). In 81 patients, AF was an incidental diagnosis (31.5%). For one female patient, the trigger for AF detection was uncertain. In symptomatic patients, palpitations were the most frequent trigger (*n* = 79; 47.3%), followed by dyspnoea (*n* = 73; 43.7%).

**Table 2:** Trigger for diagnosis of new atrial fibrillation, stratified by gender and by age group

		Gender			Age group		
Trigger	Patients with AF (*n* = 258)	Male (*n* = 120)	Female (*n* = 138)	*P*-value	65–74 years (*n* = 83)	≥ 75 years (*n* = 175)	*P*-value
*Symptoms*^a^	167 (64.7)	76 (63.3)	91 (65.9)	0.662	61 (73.5)	106 (60.6)	0.042
– Palpitations	79 (30.6)	30 (25.0)	49 (35.5)	0.068	37 (44.6)	42 (24.0)	< 0.001
– Dyspnoea	73 (28.3)	33 (27.5)	40 (29.0)	0.792	16 (19.3)	57 (32.6)	0.027
– Fatigue/malaise	40 (15.5)	16 (13.3)	24 (17.4)	0.369	13 (15.7)	27 (15.4)	0.961
– Chest pain	35 (13.6)	17 (14.2)	18 (13.0)	0.793	15 (18.1)	20 (11.4)	0.145
– Syncope/collapse	28 (10.9)	12 (10.0)	16 (11.6)	0.681	10 (12.0)	18 (10.3)	0.671
– Dizziness	28 (10.9)	13 (10.8)	15 (10.9)	0.993	10 (12.0)	18 (10.3)	0.671
– Other^b^	40 (15.5)	14 (11.7)	26 (18.8)	0.112	10 (12.0)	30 (17.1)	0.291
Stroke	9 (3.5)	5 (4.2)	4 (2.9)	0.737^d^	0	9 (5.1)	0.062^d^
Incidental^c^	81 (31.5)	39 (32.5)	42 (30.7)	0.751	21 (25.3)	60 (34.3)	0.163
Other^c^	0	0	0	NA	0	0	NA

Data are *n* (%)

*NA* not applicable

^a^Patients could have more than one symptom

^b^Other symptoms were peripheral oedema (12), nausea/vomiting (9), diarrhoea (2), transpiration (4), exercise intolerance (2), back pain (3), cough (2), unstable feeling (1), anxiety (1), agitation (1), tremor (1), blurry sight (1), heavy feeling in legs (1) and unclear (6)

^c^For one female patient, it was unclear whether the AF diagnosis was incidental or if there was another reason the AF diagnosis was made (which would change first column to *n* = 257 and third column to* n* = 137)

^d^Fisher’s exact test instead of Pearson’s chi-square test

Overall, patients aged ≥ 75 years were less often diagnosed with AF based on their symptoms than 65–74-year-olds (60.6% vs 73.5%; *p* = 0.042). In the older age group, palpitations occurred less frequently (24.0% vs 44.6%; *p* < 0.001) and dyspnoea more frequently (32.6% vs 19.3%; *p* = 0.027).

### Setting of irregular heartbeat detection

**Fig. 2:**
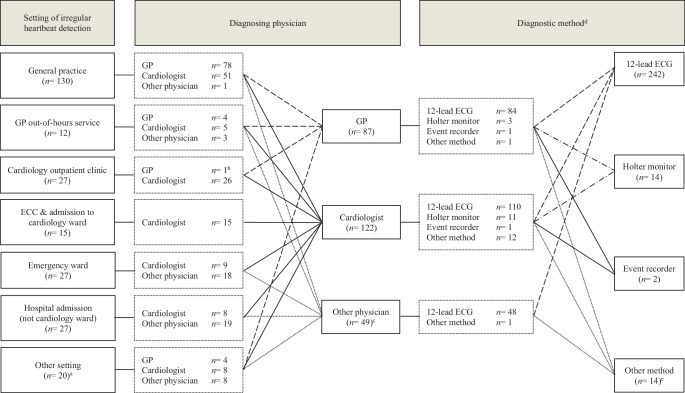
Setting where irregular heartbeat was first detected, diagnosing physician and diagnostic methods applied (*n* = 258). *ECC* emergency cardiac care department, *ECG* electrocardiogram. ^a^Other settings were: outpatient clinic other than cardiology ward (7), pre-surgical screening (4), ambulance (7), driver license bureau (1) and diabetes centre (1). ^b^Irregular heart rate of this patient was detected in a cardiology outpatient clinic abroad, after which the general practitioner (*GP*) in the Netherlands diagnosed her with AF and initiated treatment. ^c^Other physicians were: emergency physician (11), internist (9), neurologist (7), pulmonologist (4), geriatrician (4), surgeon (4), anaesthetist (2), ambulance personnel (2), intensivist (2), nephrologist (1), oncologist (1), orthopaedist (1) and unclear (1). ^d^Due to multiple answer options, observed numbers exceed 100. In 12 cases, a combination of two diagnostic methods was applied and in 1 case, three methods were applied. ^e^Other methods were: telemetry (3), pacemaker (3), cardiac stress test (3) and unclear (5)

In 142 cases (55.0%), the irregular heartbeat was detected in general practice, either during working hours or at the out-of-hours service (Fig. 2). In 42 cases (16.3%), it was first noted in the cardiology department, either at the emergency cardiac care department, during admission to the cardiology ward or at the cardiac outpatient clinic.

### Diagnosing physician and diagnostic method

In 87 patients (33.7%), AF was diagnosed by the GP, and in 122 patients (47.3%), the diagnosis was made by the cardiologist (Fig. 2). When the irregular heartbeat was detected in general practice, AF was most often diagnosed by the GP (*n* = 78; 60.0%).

Almost all diagnoses (*n* = 242; 93.8%) were based on a 12-lead ECG; the remaining 16 (6.2%) were based on ambulatory monitoring. All AF diagnoses made after stroke were based on a 12-lead ECG.

### Silent versus symptomatic AF

As previously stated, 31.5% of the patients had silent AF. We compared them with the AF patients in whom targeted diagnostics were initiated because of suspect symptoms or a stroke (symptomatic AF). Patients with silent AF were on average 2 years older than those with symptomatic AF (79.0 vs 77.0 years; *p* = 0.033) (Tab. 3). The setting of irregular heartbeat detection and the physician diagnosing AF differed between patients with silent and those with symptomatic AF (both *p* < 0.001). In most patients with symptomatic AF, the irregular heartbeat was detected in general practice, while the same was true for a small proportion of patients with silent AF (61.0% vs 27.2%; *p* < 0.001). In symptomatic AF, the diagnosing physician was more often a GP than in silent AF (39.0% vs 21.0%; *p* = 0.008).

**Table 3:** Comparison of characteristics of patients in whom diagnosis of atrial fibrillation (*AF*) was made incidentally (silent AF) versus those in whom AF diagnosis was made following investigation for suspect symptoms or stroke (symptomatic AF)

Variable	Silent AF (*n* = 81)	Symptomatic AF (*n* = 177)	*P*-value
Age in years	79.0 ± 6.8	77.0 ± 7.0	0.033
Female	42 (51.9)	96 (54.2)	0.721
*Comorbidity*	62 (76.5)	129 (72.9)	0.534
– Diabetes mellitus	24 (29.6)	44 (24.9)	0.420
– Heart failure	10 (12.3)	11 (6.2)	0.095
– Hypertension	49 (60.5)	100 (56.5)	0.546
– Previous stroke/TIA	11 (13.6)	25 (14.1)	0.907
– Thromboembolism	3 (3.7)	10 (5.6)	0.760^e^
– Vascular disease^a^	15 (18.5)	41 (23.2)	0.401
Atrial flutter^b^	7 (8.6)	16 (9.0)	0.940
*Setting of irregular heartbeat detection*^c^			< 0.001
– General practice	22 (27.2)	108 (61.0)	< 0.001
– GP out-of-hours service	1 (1.2)	11 (6.2)	0.111^e^
– Cardiology outpatient clinic	17 (21.0)	10 (5.6)	< 0.001
– ECC & admission to cardiology ward	0	15 (8.5)	0.004^e^
– Emergency ward	10 (12.3)	17 (9.6)	0.504
– Hospital admission (not cardiology ward)	20 (24.7)	7 (4.0)	< 0.001
– Other location	11 (13.6)	9 (5.1)	0.018
*Diagnosing physician*^c^			< 0.001
– GP	18 (21.0)	69 (39.0)	0.008
– Cardiologist	35 (43.2)	87 (49.2)	0.375
– Other physician	28 (34.6)	21 (11.9)	< 0.001
*Diagnostic method*^d^			
– 12-lead ECG	75 (92.6)	167 (94.4)	0.587
– Holter monitor	3 (3.7)	11 (6.2)	0.409
– Event recorder	1 (1.2)	1 (0.6)	0.530^e^
– Other method	7 (8.6)	7 (4.0)	0.123

Data are mean ± standard deviation or *n* (%)

*TIA* transient ischaemic attack, *GP* general practitioner, *ECC* emergency cardiac care department, *ECG* electrocardiogram

^a^Vascular disease includes peripheral vascular disease, myocardial infarction and angina pectoris

^b^In silent-AF group, AF classification was missing for one person (*n* = 80)

^c^For every patient, only one answer option could be selected (exclusive categories)

^d^For every patient, multiple answer options could be selected (non-exclusive categories)

^e^Fisher’s exact test instead of Pearson’s chi-square test

During admission to the cardiology ward or emergency cardiac care, no silent AF was found. Most cases of AF detected by physicians other than GPs or cardiologists were found incidentally (*n* = 28; 57%). The diagnostic methods did not differ significantly between silent and symptomatic AF.

## Discussion

### Main findings

In this cohort study, we explored the diagnostic process leading to the detection of AF in 258 patients. More than half of the diagnoses were first suspected in primary care and a sixth in the cardiology department. In two-thirds of the patients, the AF diagnosis was based on their symptoms. In 3.5% of the patients, AF detection was triggered by a stroke. In almost a third of the cases, AF was detected incidentally (silent AF). The trigger leading to the AF diagnosis did not differ for men and women. Overall, patients aged ≥ 75 years less often presented with symptoms than younger patients.

Compared with other physicians, GPs more often detected AF after targeted examination—based on the presence of suspect symptoms or a previous stroke—and less often incidentally. For cardiologists, this difference was not significant. Other physicians found most of their AF cases incidentally. GPs independently diagnosed one-third of the patients, and cardiologists diagnosed almost half of all patients. The vast majority of diagnoses was based on a 12-lead ECG, and approximately 6% was based on ambulatory monitoring.

### Trigger for AF detection

Palpitations and dyspnoea were more common symptoms than dizziness, syncope and chest pain, which is in agreement with previous findings [16, 17]. Women were more often symptomatic than men in the study by Lip et al., whereas we found no gender-related differences [16]. In another study evaluating ECGs performed in Dutch primary care, half of all new AF diagnoses were based on routine ECGs for programmatic cardiovascular care [18].

In previous studies among patients with known AF, the percentage of silent AF varied from 11 to 30% [8, 17, 19, 20]. In the study by Kerr et al., 21% of newly diagnosed AF was silent [21]. In our study, approximately a third of patients had an incidental AF diagnosis. Only 3.5% of the patients with AF was diagnosed after a stroke, compared with 4–14% in other studies [5, 22, 23]. Relatively many AF cases were detected incidentally and few after a stroke, suggesting that AF is detected at an early stage in everyday healthcare. This might explain why opportunistic screening has yielded insufficient new AF cases compared with usual care [15]. An alternative explanation is underdiagnosis of paroxysmal AF in post-stroke patients due to underuse of ambulatory monitoring [24]. However, as ambulatory monitoring is also underused in symptomatic patients [10, 11], underdiagnosis cannot fully explain the low proportion of patients diagnosed with AF after a stroke.

### Setting of irregular heartbeat detection

The irregular heartbeat was most often detected in general practice and less often in secondary care. This finding reflects the role of the GP as the gatekeeper in the Netherlands, where a referral is needed for a specialist consultation [25].

In a quarter of the patients, the irregular heartbeat was detected during hospital admission or emergency room visit. There, an ECG is often performed or the heart rate is monitored, creating opportunities to detect AF. Furthermore, other medical conditions for which a hospital visit may be required, such as anaemia, myocardial infarction or fever, can trigger AF [12].

### Diagnostic method

Almost all AF diagnoses in our study were based on a 12-lead ECG. This finding is in accordance with AF guidelines, which recommend a 12-lead ECG or rhythm strip showing AF for ≥ 30 s to make the diagnosis [12, 26]. Ambulatory monitoring of variable duration, depending on symptom frequency, is recommended to detect paroxysmal AF [12, 26]. In this study, few diagnoses were based on ambulatory monitoring (6.2%), which is in accordance with previous research [10, 11].

### Strengths and limitations

To our knowledge, this is the first study exploring the manner in which AF is detected in Dutch everyday healthcare. Our study has several strengths. First, we could prospectively include a substantial group of patients with newly discovered AF because of the large study cohort. Second, this cohort consisted of patients without known AF and was established by taking a random sample, thereby avoiding selection bias. Third, we did not merely rely on automated extraction of ICPC codes to confirm the AF diagnosis. Instead, we manually reviewed electronic medical records and searched for related ICPC codes to account for incorrect registrations, increasing the validity of our data. Fourth, participating practices were distributed throughout the Netherlands, which increased the generalisability of the results.

Our study also has some limitations. We were dependent on the quality and completeness of the medical records. Based on these records we could not reliably distinguish between paroxysmal, persistent and permanent AF. Furthermore, we were not able to access 27 medical files of patients with AF. As the study cohort consisted of the control arm of a trial on AF detection, participation in this trial may have influenced usual care due to a higher awareness of AF among the healthcare professionals. We aimed to reduce this influence by using a blind and stratified randomisation, prohibiting participation in other screening initiatives, blinding practices for the selected patients and offering an opt-out option instead of asking for written informed consent.

### Implications

It is vital that GPs, who fulfil the gatekeeper role, know what signals to look for and when to suspect AF, as they are often the first physicians who encounter patients with new AF. Specialists other than cardiologists also have to be vigilant, as silent AF was shown to represent a substantial portion of AF cases. Implementation of local working agreements and close cooperation between primary and secondary care and between specialists should facilitate the diagnostic process.

## Conclusion

Diagnosing AF is a multidisciplinary process, in which not only cardiologists are involved but also GPs and other physicians. While an irregular heartbeat was most often first noted in general practice, cardiologists most often diagnosed AF. One-third of the patients had silent AF. Ambulatory monitoring was responsible for only a small proportion of the diagnoses made.

## Supplementary Information

**Fig. S1** Geographic distribution of 49 general practices participating in the study
