# Supplementary figures and images for: How is atrial fibrillation detected in everyday healthcare? Results of a Dutch cohort study
Source: Neth Heart J. 2022 Sep 1;31(2):76–82. doi: 10.1007/s12471-022-01719-2 (PMC9892390; doi:10.1007/s12471-022-01719-2)

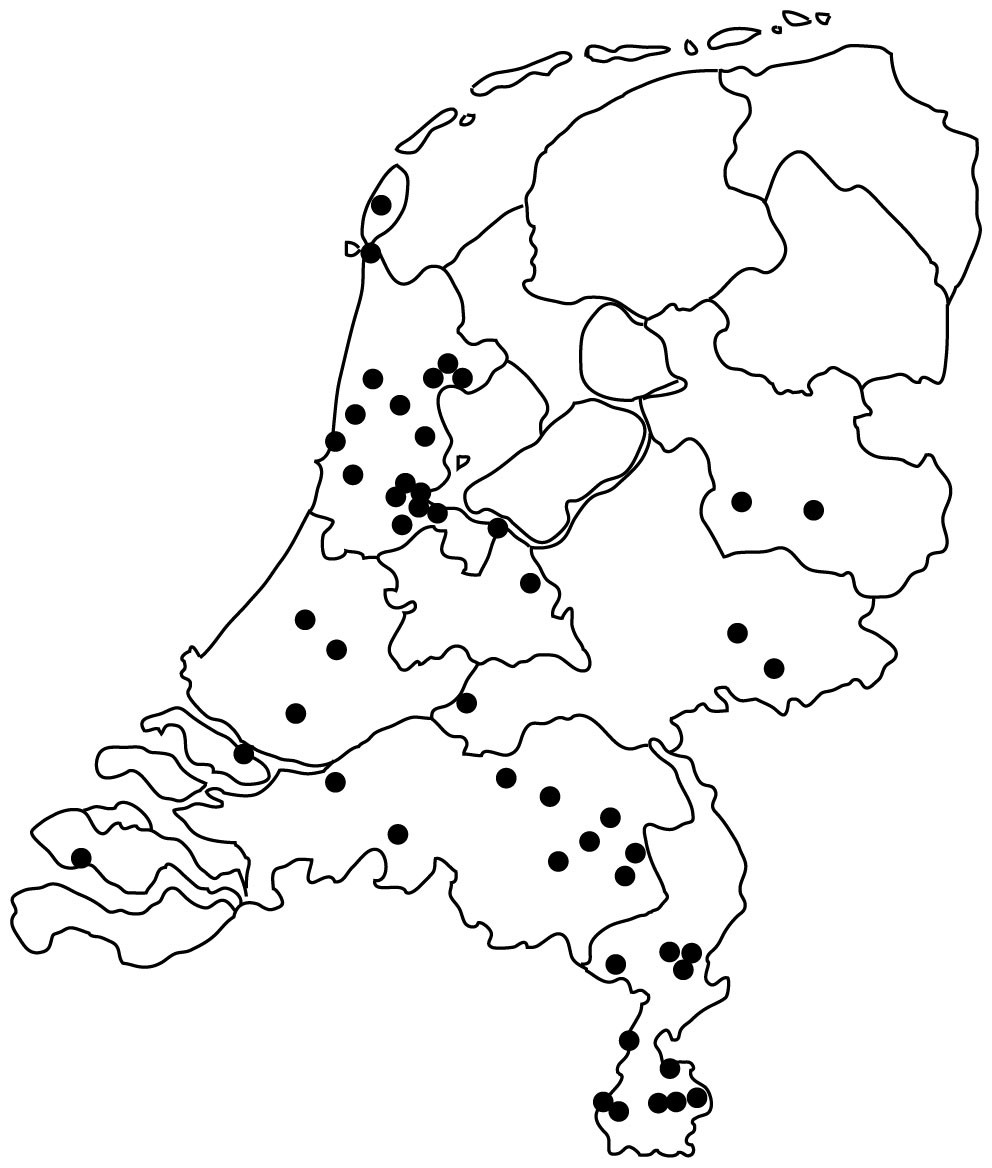

Supplement: Supplementary file 1 — Fig. S1 Geographic distribution of 49 general practices participating in the study [file 12471_2022_1719_MOESM1_ESM.jpg]
